# Gram-Negative Bacterial Lipopolysaccharide Stimulates Activin A Secretion from Human Amniotic Epithelial Cells

**DOI:** 10.1155/2013/789012

**Published:** 2013-07-17

**Authors:** Yumiko Abe, Risa Marukawa, Nami Tsuru, Maki Sato, Hiroko Matsuda, Hisanobu Sadakata, Takashi Kameda, Takashi Minegishi

**Affiliations:** ^1^Department of Laboratory Sciences, Graduate School of Health Sciences, Gunma University, 3-39-22 Showa, Maebashi, Gunma 371-8514, Japan; ^2^Kuki General Hospital, Kuki, Saitama 346-0021, Japan; ^3^Miyazaki Prefectural Nobeoka Hospital, Nobeoka, Miyazaki 882-0835, Japan; ^4^Yokota Maternity Hospital, Maebashi, Gunma 371-0031, Japan; ^5^Department of Obstetrics and Gynecology, Graduate School of Medicine, Gunma University, 3-39-22 Showa, Maebashi, Gunma 371-8511, Japan

## Abstract

Activin A is involved in inflammation. The present study was performed to clarify if lipopolysaccharide, a component of Gram-negative bacteria, stimulates activin A secretion from human amniotic epithelial cells and to determine if activin A plays a role in amnionitis. Fetal membranes were obtained during elective cesarean sections performed in full-term pregnancies of patients without systemic disease, signs of premature delivery, or fetal complications. Amniotic epithelial cells were isolated by trypsinization. The activin A concentrations in the culture media were measured by enzyme-linked immunosorbent assay, and cell proliferation was assessed by 5-bromo-2′-deoxyuridine incorporation. Amniotic epithelial cells secreted activin A in a cell density-dependent manner, and lipopolysaccharide (10 **μ**g/mL) enhanced the secretion at each cell density. Lipopolysaccharide (10–50 **μ**g/mL) also stimulated activin A secretion in a dose-dependent manner. Contrary to the effect of activin A secretion, lipopolysaccharide inhibited cell proliferation in amniotic epithelial cells. The present study suggests that lipopolysaccharide stimulation of activin A secretion may be a mechanism in the pathogenesis of amnionitis.

## 1. Introduction

Activins, which were first identified as stimulators of FSH secretion, are pluripotent growth factors in the TGF-beta superfamily [1]. Among the many functions of activins, the involvement of activin A in inflammation has been noted [1–6]. The administration of lipopolysaccharide (LPS), a Gram-negative bacterial cell wall component, prominently increases the serum activin A level in sheep and mice [7, 8]. Activin A levels in the circulation were higher in mice that died than in mice that survived after the administration of a sublethal dose of LPS [8]. Furthermore, cotreatment with follistatin, which neutralizes activin by binding to it, increased the survival rate of LPS-treated mice [8]. The activin A release in mice depends on a signaling cascade through Toll-like receptor 4 (TLR4), a receptor for LPS [8].

Serum activin A concentrations are elevated in patients with septicemia [9]. During pregnancy, serum activin A concentrations increase [10] and activin A dimers and activin beta-A subunits are detected in trophoblasts and amniotic epithelial cells (AEC) in the human placenta [11, 12]. The expression of activin beta-A subunit mRNA in fetal membranes increases during labor [13]. Activin A concentrations in amniotic fluid are higher in women with preterm labor than in women without preterm labor at the same stage of gestation [13]. Activin A concentrations in amniotic fluid are also elevated in women with intra-amniotic infection [14].

The notion that activin A is involved in chorioamnionitis is also supported by *in vitro* studies. Activin A secretion from gestational tissues and cells is stimulated by inflammatory cytokines. Tumor necrosis factor-*α* (TNF-*α*) stimulates activin A production in explant cultures of human amnion, and choriodecidua [15], and cultured human AEC [16]. Interleukin (IL)-1*β* also stimulates activin A production in the amnion, choriodecidua [15], and AEC [16]. Activin A modulates the secretion of IL-6, IL-8, and prostaglandin E2 in explant cultures of human amnion and choriodecidua [17].

Compared to the effects of TNF-*α* and IL-1*β*, the effect of LPS on activin A secretion from human AEC is inconsistent, despite the expression of functional TLR4 in human AEC [18]. Rosenberg et al. reported that LPS stimulated activin A release from cultured amniochorion explants but not from placental villous tissue [14]. On the other hand, Keelan et al. reported that LPS did not affect activin A secretion from amnion explant cultures [15]. Since the amnion is an avascular tissue and AEC are located in the innermost layer of the amnion, secretions of AEC must be directly released into the amniotic fluid and affect the fetus. The present study was performed to determine if LPS stimulates activin A secretion from AEC and to verify the notion that activin A is involved in amnionitis.

## 2. Materials and Methods

### 2.1. Reagents

LPS that was phenol extracted from *E. coli* O26 was purchased from Paesel & Lorei GmbH (Hanau, Germany) (catalogue no. 100976, Lot. 15303).

### 2.2. AEC Culture

With the permission of the Institutional Review Board of Gunma University Hospital and the written informed consent of the patients, we obtained fetal membrane samples during elective cesarean sections performed on four patients with full-term pregnancies who did not have any systemic disease, signs of premature delivery, or fetal complications. AEC were prepared as previously reported [19] on the basis of the method established by Okita et al. [20] with slight modifications. Briefly, the chorion was removed from amnion mechanically, and the amnion was washed thoroughly with phosphate-buffered saline. The removal of the chorion was ascertained by using hematoxylin/eosin-stained paraffin sections of amnions. The amniotic membrane was cut into pieces and incubated in 170 mL of Krebs-Ringer solution containing 0.15% trypsin, 1.26 mg/mL sodium bicarbonate, 25 mM HEPES, 100 *μ*g/mL streptomycin, and 0.5 *μ*g/mL amphotericin B at 37°C in a spinner flask. The liberated cells were decanted at 30 min intervals, and the incubation was performed seven times with freshly made trypsin solution. Each fraction of dispersed cells was centrifuged and resuspended in DME/Ham's F12 medium supplemented with 10% fetal bovine serum (FBS), 100 *μ*g/mL streptomycin, and 0.5 *μ*g/mL amphotericin B. The first fraction was discarded. The cell viability of the remaining fractions was determined by trypan blue exclusion, and the fractions with viabilities of at least 80% were pooled. Cells in 200 *μ*L of medium were seeded in each well of 96-well plates and cultured in a humidified atmosphere containing 5% CO_2_-95% air at 37°C. These cells were used for measuring activin A and cell proliferation.

### 2.3. Activin A ELISA

Activin A concentrations in culture medium were measured by using an activin A assay kit (Oxford Bio-Innovations, Oxfordshire, UK) according to the manufacturer's instructions. The samples, which were pretreated with sodium dodecylsulfate and hydrogen peroxide, were added along with assay diluent to microwells coated with a monoclonal antibody specific for the beta-A subunit of activin. After 1 h of incubation, biotinylated monoclonal antibody for the beta-A subunit of activin was added and incubated overnight. The following day, wells were washed and streptavidin-alkaline phosphatase solution was added. After 1 h of incubation, the wells were washed and incubated with substrate solution for 2 h. Then, amplifier solution was added, and the absorbance at 490 nm was measured.

### 2.4. Cell Proliferation Assay

Cell proliferation was measured by assessing 5-bromo-2′-deoxyuridine (BrdU) incorporation by using a Cell Proliferation ELISA, BrdU (colorimetric) kit (Roche Diagnostics, Mannheim, Germany). The assay was performed in accordance with the manufacturer's instructions. Cells were seeded into 96-well microplates with or without LPS. After a 96 h incubation, 20 *μ*L of 100 *μ*M BrdU solution was added to each well containing 200 *μ*L of medium, and the cells were reincubated for another 24 h. The culture medium was removed, the cells were fixed, and the DNA was denatured. The cells were then incubated with mouse anti-BrdU monoclonal antibody conjugated to peroxidase at room temperature for 90 min. After removal of the antibody, the immune complexes were detected by subsequent reaction with tetramethylbenzidine. The reaction was stopped by the addition of sulfuric acid, and the product was quantified by measuring the absorbance at 450 nm.

### 2.5. Statistics

The data from quadruplicate cultures are presented as the mean ± SE. Comparison between groups was performed by using one-way ANOVA, and the significance of the differences between the mean values was tested by using Fisher's PLSD test. Comparison between two groups was performed by using the Student's *t*-test. *P* values < 0.05 were considered statistically significant.

## 3. Results

**Figure 1:**
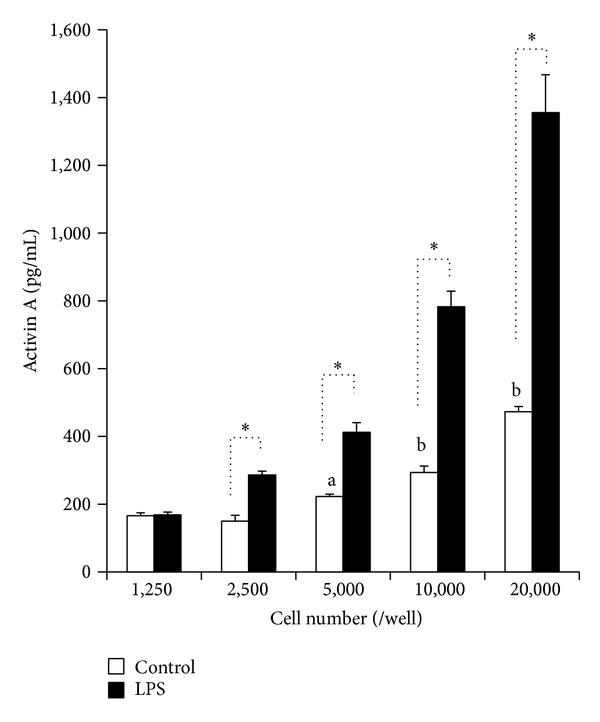
Effect of LPS on activin A secretion at various densities of AEC. Human AEC were seeded in 96-well microplates at densities of 1,250, 2,500, 5,000, 10,000, and 20,000 cells per well in 200 *μ*L of DME/Ham's F12 medium supplemented with 10% FBS, 100 *μ*g/mL streptomycin, and 0.5 *μ*g/mL amphotericin B. Either LPS (10 *μ*g/mL) or vehicle (control) was added to each well. After 96 h of culture, activin A in the medium was measured. Closed rectangles: LPS. Open rectangles: control. The results from the quadruplicate assay are shown as the mean ± SE (*n* = 4). ^a^ *P* < 0.05 compared with the control value at 1,250 cells/well. ^b^ *P* < 0.01 compared with the control value at 1,250 cells/well. *P < 0.01 compared with the control value at each cell density.

Variable densities of AEC were seeded into 96-well plates and incubated. After 96 h of incubation, activin A concentrations in the media were measured. Increased activin A concentrations in the media were observed in AEC without LPS in a cell density-dependent manner, and the activin A concentrations were significantly higher at cell densities of 5,000 cells/well or higher (*P* < 0.05) (Figure 1). LPS (10 *μ*g/mL) significantly increased activin A concentrations at densities of 2,500 cells/well and greater (*P* < 0.01). Activin A concentrations of cell lysates from either LPS-stimulated AEC or control AEC were below assay sensitivity (data not shown). Therefore, the increase of activin A concentrations in the medium was equivalent to the increase of activin A production and secretion in AEC.

**Figure 2:**
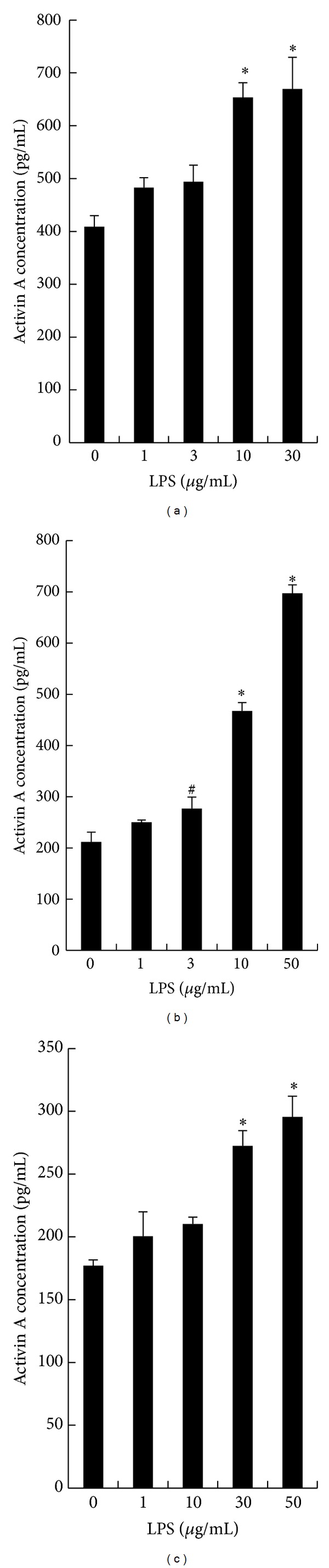
Dose-dependent effects of LPS on activin A secretion in three independent experiments. Fetal membranes were obtained from three patients, and AEC from each patient were cultured independently at a density of 20,000 cells per well in DME/Ham's 12 medium supplemented with 10% FBS, 100 *μ*g/mL streptomycin, 0.5 *μ*g/mL amphotericin B, and various concentrations of LPS for 96 h. Each independent result is shown in panels (a), (b), and (c). The results are shown as the mean ± SE (*n* = 4). ^#^ *P* < 0.05 compared with the control (LPS; 0 ng/mL) value. *P < 0.01 compared with the control (LPS; 0 ng/mL) value.

The stimulatory effects of various concentrations of LPS on activin A secretion in AEC were confirmed by three independent studies of AEC from three patients. In each experiment, a dose-dependent increase in activin A secretion from AEC occurred after 96 h of culture with LPS (Figure 2).

**Figure 3:**
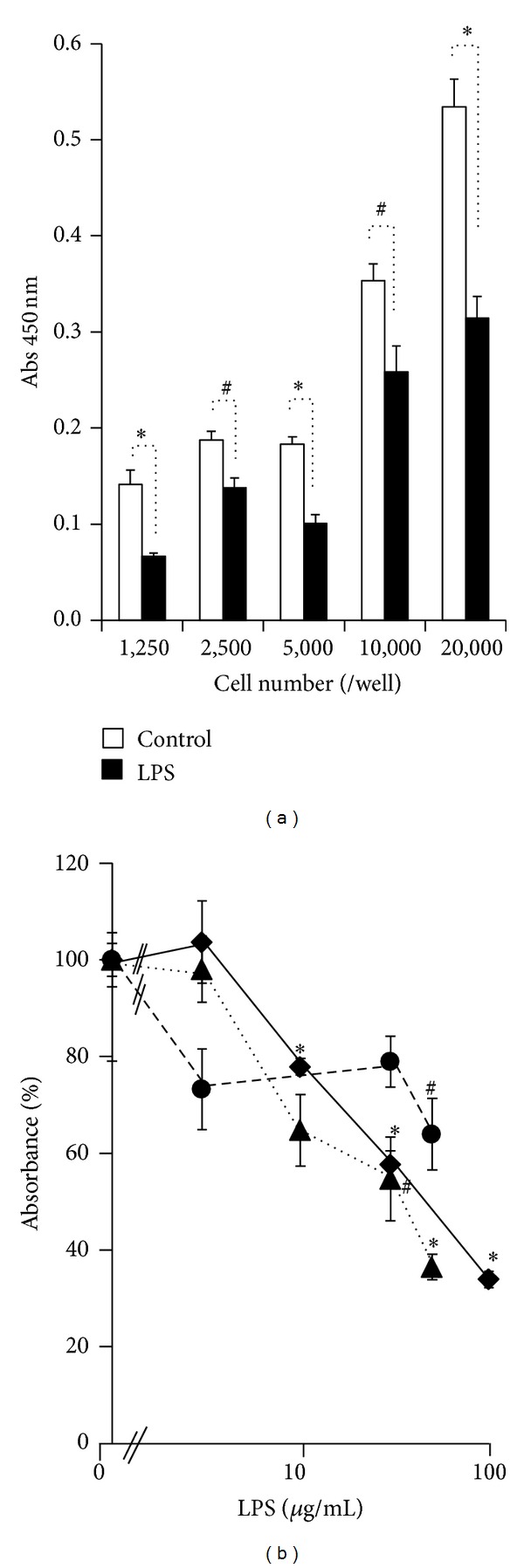
Effect of LPS on BrdU incorporation in AEC. (a) AEC were seeded in 96-well microplates at densities of 1,250, 2,500, 5,000, 10,000, and 20,000 cells per well in 200 *μ*L of DME/Ham's F12 medium supplemented with 10% FBS, 100 *μ*g/mL streptomycin, and 0.5 *μ*g/mL amphotericin B. Either LPS (10 *μ*g/mL) or vehicle (control) was added to each well. After 96 h of culture, BrdU incorporation was studied. The results from the quadruplicate assay are shown as the mean ± SE (*n* = 4). ^#^ *P* < 0.05 compared with the control value at each cell density. *P < 0.01compared with the control value at each cell density. (b) Fetal membranes were obtained from three patients. AEC from each patient were cultured independently in DME/Ham's 12 medium supplemented with 10% FBS, 100 *μ*g/mL streptomycin, 0.5 *μ*g/mL amphotericin B, and various concentrations of LPS. After 96 h of culture, BrdU incorporation was studied. Closed circles, closed rectangles, and closed triangles show AEC from each patient. The absorbance at AEC cultured without LPS is shown as 100%. The results from the quadruplicate assay are shown as the mean ± SE (*n* = 4). ^#^ *P* < 0.05 compared with the control (LPS; 0 ng/mL) value. *P < 0.01 compared with the control (LPS; 0 ng/mL) value.

The effects of LPS on AEC proliferation were also studied. LPS (10 *μ*g/mL) suppressed cell proliferation at each cell density (1,250–20,000 cells/well) (Figure 3(a)). A dose-dependent inhibitory effect of LPS on AEC proliferation was also shown by three independent studies that used AEC from three patients (Figure 3(b)).

## 4. Discussion

AEC secreted activin A in a cell density-dependent manner in cultures of AEC prepared from the trypsinization of amnions from women with full-term pregnancies. This type of AEC culture has been utilized in studies of the syntheses and secretion of phospholipids and prostaglandins [20, 21], matrix metalloproteinase-9 and extracellular matrix metalloproteinase inducer [22, 23], brain natriuretic peptide [24], endothelin-1 [24, 25], fibronectin [26, 27], albumin and glycogen [28], and cystic fibrosis transmembrane conductance regulator [29]. Keelan et al. used this method to study activin A production by AEC [16]. The cell density and incubation time of our AEC cultures were comparable (although not identical) to the conditions used by Keelan et al. 

LPS enhanced the activin A secretion at each cell density (2,500–20,000 cells/well). LPS also stimulated activin A secretion dose dependently in three independent cultures of AEC from three patients. Rosenberg et al. reported that LPS stimulated activin A release from cultured amniochorion explants but not from placental villous tissue [14]. Our study has clearly shown that LPS stimulates activin A secretion from AEC. On the other hand, Keelan et al. reported that LPS did not affect activin A production in amnion explant cultures [15]. The components of amniotic explants other than AEC might inhibit the effects of LPS, or the LPS dose used in their study (5 *μ*g/mL) might not be sufficient to stimulate activin A secretion. In a mouse epithelial Sertoli cell line, the secretion of activin A is enhanced through TLR4 by LPS stimulation [8]. Since functional TLR4 is expressed in human AEC [18], future studies must determine if similar mechanisms affect activin A secretion by LPS-stimulated AEC. 

LPS stimulated activin A secretion from AEC at doses of 10 *μ*g/mL or higher. The endotoxin concentrations in the amniotic fluid of women with premature rupture of membranes were between several hundred pg/mL to several *μ*g/mL, as determined by the Limulus amebocyte lysate assay with *E.coli *LPS as the standard [30]. Compared to the endotoxin concentrations in the amniotic fluid, the LPS doses that stimulated activin A secretion from AEC were higher. On the other hand, the LPS doses used in the present study are comparable to the doses used in previous studies of human gestational tissues and cells [31–35]. Local LPS concentrations in the microenvironment of AEC must be higher than the concentrations in amniotic fluid when Gram-negative bacteria invade the amnion [36].

LPS suppressed cell proliferation. Therefore, the increase of activin A secretion is caused by enhanced production and secretion of activin A in AEC rather than an increased number of AEC. LPS induces apoptosis directly or indirectly in several types of cells [37–41]. Apoptosis of AEC occurs in fetal membranes from patients with chorioamnionitis [42]. The present results are in accordance with these findings. The tensile strength of fetal membranes is provided by collagens in the amnion, and the tensile strength is reduced in chorioamnionitis by the degradation of collagens by matrix metalloproteinases [43]. LPS itself might weaken the strength of membranes by suppressing AEC proliferation.

In conclusion, LPS stimulated activin A secretion from human AEC, which may be a mechanism in the pathogenesis of amnionitis.
